# The contribution of human papilloma virus infection to cutaneous squamous cell carcinoma in patients with chronic lymphocytic leukemia

**DOI:** 10.1002/jha2.181

**Published:** 2021-03-04

**Authors:** Preeya Bhavsar‐Bhakta, Mugahed Hamza, Sepideh Mehravaran, Bhuvaneswari Krishnan, Qin He, Steven Tyring, Peter Rady, Gustavo Rivero, Daniel N. Cohen, Iberia Romina Sosa

**Affiliations:** ^1^ Department of Medicine, Baylor College of Medicine Houston Texas USA; ^2^ Department of Pathology & Immunology, Baylor College of Medicine Houston Texas USA; ^3^ The Dan L. Duncan Comprehensive Cancer Center at Baylor College of Medicine Houston Texas USA; ^4^ Department of Dermatology McGovern Medical School Houston Texas USA; ^5^ Bristol Myers Squibb Lawrenceville New Jersey USA; ^6^ Department of Hematology and Oncology, Fox Chase Cancer Center Philadelphia Pennsylvania USA

**Keywords:** chronic lymphocytic leukemia (CLL), cutaneous squamous cell carcinoma, human papilloma virus (HPV)

## Abstract

Patients with chronic lymphocytic leukemia (CLL), a B‐cell malignancy characterized by impaired humoral and cellular immunity, are at increased risk of developing cutaneous squamous cell carcinoma (cSCC). Human papilloma virus (HPV) is the most common sexually transmitted infection worldwide and it has been associated with various malignancies, including cSCC. Impaired cell‐mediated immunity is considered a primary risk factor in HPV‐induced cSCC. We examined cSCC lesions from CLL patients with consensus review and HPV genetic analysis to further characterize the relationship between HPV and prevalence of cutaneous malignancy in this population. Eleven patients with CLL contributed 35 cSCCs. Treatment with chemotherapy shortened the latency time to first cSCC. HPV was detected in 54% of the lesions. Among the HPV‐positive cSCC lesions, 84% of the lesions contained alpha‐genus HPV, 42% contained beta‐genus HPV, and 26% of the lesions contained both genera. There was a significant association between HPV‐containing lesions and peritumoral lymphocytic inflammation, suggesting this as a future area for further characterization. The majority of the lesions, including those with alpha‐genus HPV, occurred in sun‐exposed areas, such as the scalp and face. These findings may lead to practice‐changing recommendations for skin cancer, including the use of vaccinations to reduce HPV‐associated skin cancer.

## 1 INTRODUCTION

Chronic lymphocytic leukemia (CLL) is the most common adult leukemia in the United States [1]. CLL is a B‐cell lymphoproliferative disorder that follows an indolent clinical course, with a majority of patients remaining asymptomatic for several years. However, a diagnosis of CLL is associated with an increased risk of developing secondary malignancies, with the most common being non‐melanoma skin cancers (NMSCs), including cutaneous squamous cell carcinoma (cSCC). Interestingly, patients with CLL are reported to have a 3.7‐fold increased incidence of NSMC and a 17‐fold increased risk in NSMC‐associated mortality when compared to their non‐CLL counterparts [2, 3, 4].

Although the incidence of cSCC among CLL patients is well established, the clinical features predisposing this population to higher risk of NMSC remain poorly characterized. A recent study from Mayo Clinic examined a large CLL cohort and established that the strongest risk factor for developing SCC was a prior history of any skin cancer [5]. CLL‐international prognostic index (IPI) was independently associated with increased risk of cSCC; patients with more aggressive disease at the time of CLL diagnosis had a higher risk for developing cSCC. Interestingly, patients with high risk (HR) CLL but no prior history of NMSC had better outcomes compared to those individuals with prior NMSC history regardless of CLL‐IPI status, underscoring the importance of prior skin cancer in future risk of cSCC for CLL patients.

In cervical carcinoma and squamous cell carcinoma (SCC) of the oropharynx and larynx, human papilloma viruses (HPVs) of the alpha‐genus, most frequently, HPV‐16, ‐18, ‐31, and ‐33, drive carcinogenesis and are categorized as HR‐HPV subtypes. HPVs found in cutaneous epithelial surfaces are most often of the beta and gamma genera. There is strong evidence demonstrating a central role for beta genus HPV, specifically HPV‐5 and HPV‐8, in skin cancer risk of immunosuppressed individuals, specifically via coincident mutation burden, including UV‐signature mutations [6] and activation of aberrant proliferation pathways [7]. This pathophysiology has been well documented in solid organ transplant recipients treated with immunosuppressive therapy [8] and patients with somatic mutations in epidermodysplasia verruciformis (EVER1/2) pathways tumor suppressor genes. CLL patients exhibit immune deficiency similar to that described in transplant patients [9, 10]. Hence, it has been suggested that HPV may play a significant role in the pathophysiology of cSCC in CLL patients through similar pathways.

The purpose of this study was to further characterize the role of HPV in the incidence of cSCC in CLL patients. We hypothesized that CLL patients with cSCC would harbor cutaneous HPV and the lesions would be morphologically unique to those of immunocompetent patients.

## 2 METHODS

### 2.1 Study population

A retrospective cohort study was conducted at the Michael DeBakey Veterans Administration Medical Center (MEDVAMC) in Houston, TX, in accordance with institutional review board (H‐24175 Baylor College of Medicine and MEDVAMC Research Committee). We queried both VISTA pathology and CPRS clinical records systems from 2013 to 2018. The search included patients with specimens for diagnosis of cSCC as well as CLL/small lymphocytic leukemia (SLL). The clinical diagnosis of CLL identified by SNOMED search was confirmed by board‐certified hematologists and included medical chart review of clinical parameters, laboratory data, and treatment history. The original cohort of CLL patients included 40 subjects. In total, 11 subjects with both CLL/SLL, and one or more cSCC, for which there was sufficient tissue to perform molecular studies, were identified. Further analysis was performed on previously procured diagnostic tissue samples that met the predefined enrollment criteria as described above.

### 2.2 Consensus dermatopathology pathology review

Histopathologic review of conventional hematoxylin & eosin (H&E) stained sections of formalin‐fixed paraffin‐embedded (FFPE) sections, 4–6 micrometers thickness, was performed under conventional light microscopy.

Each case was independently reviewed, and findings were tabulated by two pathologists (A & B) who were blinded to each other's review as well as previously rendered clinical and pathologic diagnoses. The two reviews were compared by a project manager and if results were concordant, the first interpretation was utilized. If the reviews were discordant, a third pathologist reviewed sample while blinded to the original two interpretations. The third interpretation was used with consensus review to achieve the final study interpretation.

During review, cSCC cases were examined for the specific morphologic characteristic of cSCC, presence of features of previously described HPV infection in cSCC [7], as well as features of chronic inflammation. Additionally, all cases were examined for histomorphologic features ascribed to MCPyV, HPyV6/7, and TSPyV infection [11, 12, 13]. Inflammatory changes were annotated as the presence of lichenoid, intraepithelial, and/or dermal lymphocytic infiltration (mild, moderate, or dense).

### 2.3 Cutaneous virus DNA assays

DNA was extracted from the FFPE samples utilizing Gentra Puregene Tissue Kit (Qiagen). Polymerase chain reactions (PCRs) for HPV [14], beta‐globin reference gene [7], FAP [15], PGMY‐GP5/6+ [16], and MCPyV [17] were performed based on published methods. PCR conditions were the following: denaturation, 1 min at 95^o^C; annealing, 1 min at 63^o^C; extension, 1‐minmin at 72^o^C, and 40 cycles were applied. The PCR products were separated by agarose gel electrophoresis, visualized by ethidium bromide staining and UV light. The obtained putative PCR products were purified, cloned, and sequenced. The sequencing data were evaluated by computer‐assisted analysis using NCBI‐BLAST program.

### 2.4 Statistical analysis and visualization

Tumor and viral features associated with cSCC in CLL patients were examined using Chi Square and Fisher's exact test, as appropriate. Analyses were conducted using SAS software, version 9.4 (SAS Institute Inc., Cary, NC).

## 3 RESULTS

**FIGURE 1:**
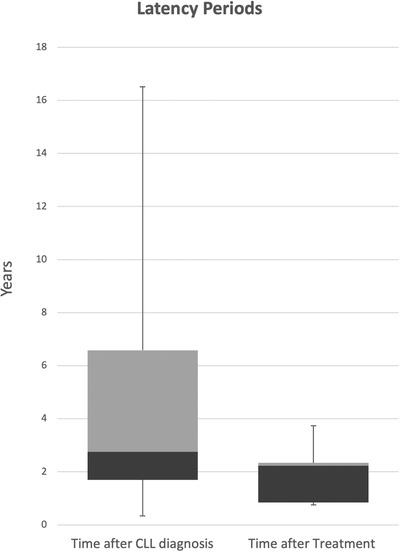
Chemotherapy for CLL reduced the latency period to the development of cSCC. Six out of 11 subjects received chemotherapy. Of these subjects, all except one developed cSCC after starting therapy. A single subject was excluded from the analysis as latency was noted to be 33 years and considered an outlier. Mean latency time from CLL diagnosis to cSCC was 5.02 years (range 0.3–16.5 years), with chemotherapy shortening this latency to 1.98 years (0.74–3.74 years)

Among the 11 CLL patients included in the study, the median age at diagnosis was 64 (range 38–87 years old); 100% were Caucasian, 100% were male, and 18% had advanced stage disease as determined by Rai criteria. Mean latency time from CLL diagnosis to cSCC was 5.02 years (range 0.3–16.5 years), after exclusion of a single patient whose latency was 33 years (> 3 SD from median of remaining cohort). Patients who received chemotherapy exhibited a shorter latency to first cSCC, approximately 1.98 years (range 0.74–3.74 years; Figure 1). Of the five patients who received treatment, one received Bruton tyrosine kinase (BTK) inhibitor, one received anti‐CD20 monotherapy, and three received a combination of anti‐CD20/purine analogue (Table 1).

**TABLE 1:** Demographic characteristics of CLL patients

		*N*
Gender	Male	11
	Female	0
Race	Caucasian	11
	Non‐Caucasian	0
Total number of SCC by patient	1	5
	2	3
	> 5	2
	> 10	1
Chemotherapy	None	6
	Anti‐CD20	4
	T‐cell suppression	3
	BTK inhibitor	1
Rai stage	Stage 0	7
	Stage 1	2
	Stage 2	
	Stage 3	1
	Stage 4	1
Age	<50	1
	51–60	3
	61–70	4
	71–80	1
	81–90	2

**FIGURE 2:**
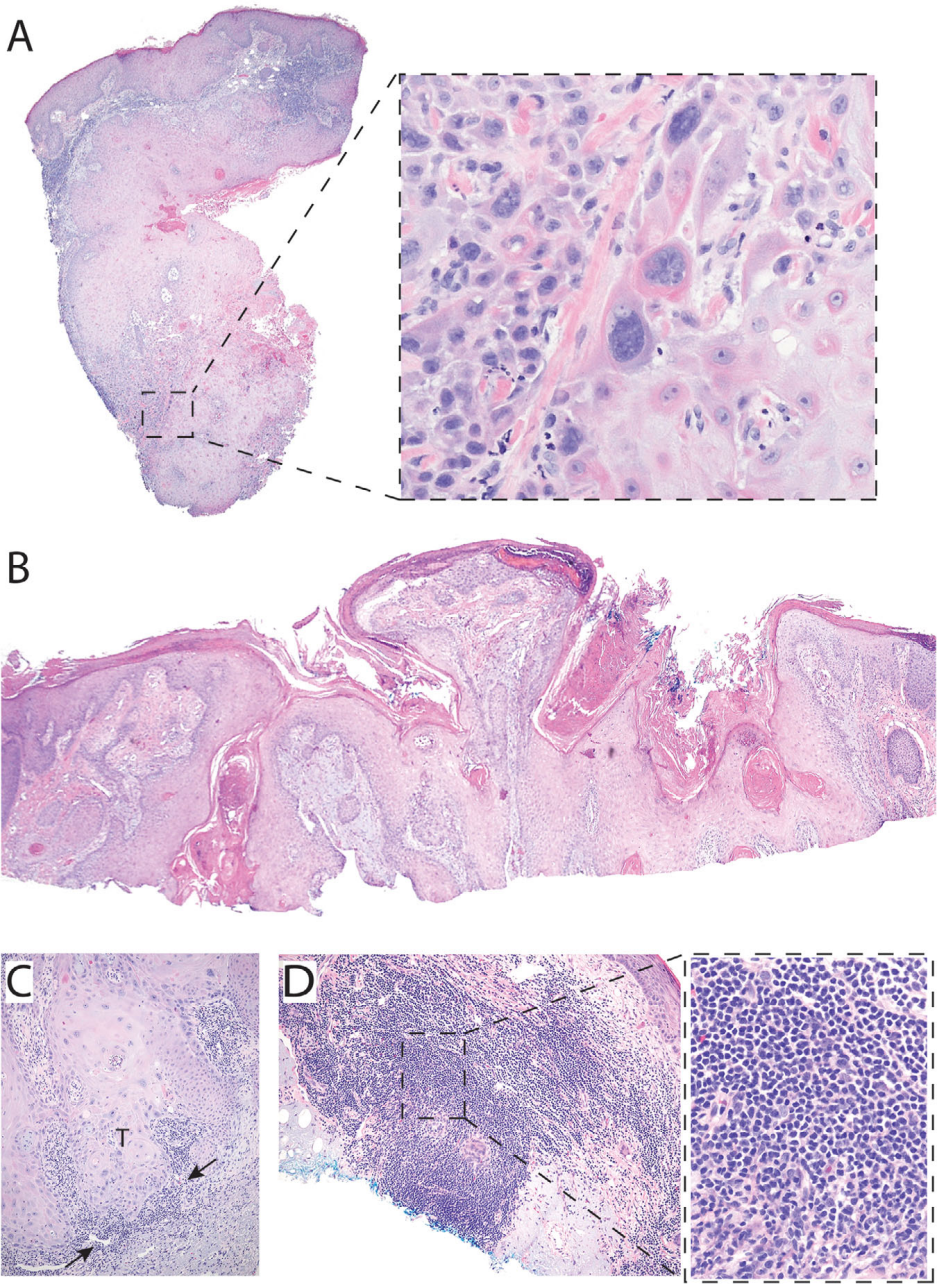
Histomorphologic characterization of cSCC lesions found in CLL patients. (A) Cutaneous SCC with markedly atypical malignant cells invading the deep dermis characteristic of conventional SCC. (H&E composite, 2x objective magnification [obj. mag.]; inset 40x). (B) SCC with papillomatosis, hyperkeratosis, focal parakeratosis, and focal hypergranulosis––consistent with wart‐like features (cSCC‐wf) H&E composite, 4x obj. mag. (C and D) Dermal lymphocytic infiltration seen in cSCC lesions of CLL patients: cSCC tumor ("T") with peritumoral small uniform lymphocytes with mild to moderate atypia suggestive of coincident CLL/SLL (arrows), (C: H&E, 20x obj. mag. Tumor‐adjacent early germinal center formation (D: H&E, 10x obj. mag; inset: H&E, 40x obj. mag.)

Most of the lesions found in our cohort were characterized as conventional cSCC (conv‐cSCC), with 8% of these displaying keratoacanothomatous cSCC features (cSCC‐KA). Briefly, the conv‐cSCCs (Figure 2A) were composed of a proliferation of atypical keratinocytes with enlarged hyperchromatic and irregular nuclei, with focal mitotic activity, expanding and filling the epidermis, with invasion of the atypical epithelial cells below the dermoepidermal junction into the dermis. Those with keratoacanthomatous features (cSCC‐KA) displayed an atypical proliferation of keratinocytes with crateriform architecture and additional glassy eosinophilic cytoplasm with tumor invasion into the dermis. The remaining 31% lesions were cSCC with wart‐like features ([cSCC‐wf]; Figure 2B), characterized by conventional cSCC features, as previously described, along with features typically seen in verrucae (warts); namely, papillomatosis, acanthosis, hyperkeratosis, hyperparakeratosis, hypergranulosis, and occasionally viral cytopathic nuclear features. Sixty percent of the lesions displayed moderate to high lymphocytic inflammation composed of small, round, uniform lymphocytes (Figure 2C), cuffing the invasive border of the SCC consistent with co‐occurrence of cutaneous involvement of CLL and SCC.

**FIGURE 3:**
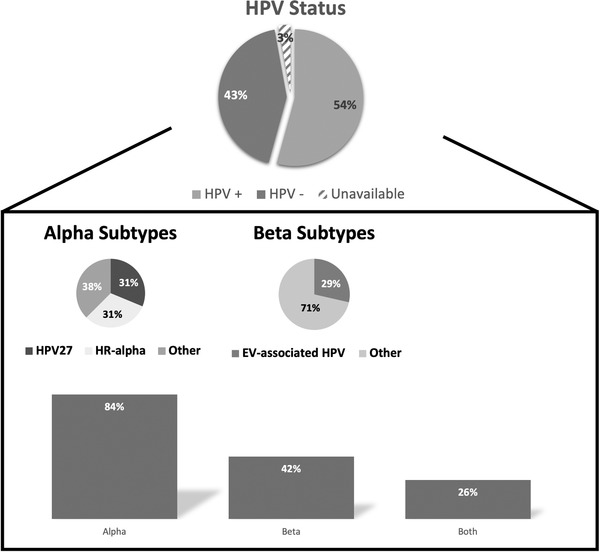
HPV status and genus association by lesion. Eleven patients with CLL contributed 35 cSCC biopsies. Fifty‐four percent of the lesions were positive for HPV, with the majority of them containing alpha‐genus subtype, 84% of HPV‐positive lesions. Thirty one percent of the lesions contained HR‐alpha, including HPV‐16 and HPV‐18, 31% contained HPV‐27, and 38% contained other alpha‐genus subtypes. Forty‐two percent of the biopsies contained beta‐genus HPV. EV‐associated HPVs, including HPV‐5, were infrequent, 29% of the lesions. Three percent of the lesions biopsied could not be genotyped due to poor sample DNA

Tumor samples from 11 CLL patients (corresponding to 35 cSCC tumors) were submitted for viral DNA genotyping. Fifty‐four percent of the submitted samples were positive for at least one HPV virus subtype. Of these, 84% of samples were positive for alpha‐genus, which include HR‐HPV‐16/18 and verrucae‐associated HPV‐27. Forty‐two percent of the HPV‐positive lesions expressed beta‐genus HPV, including epidermodysplasia verruciformis (EV)‐associated HPV‐5. Twenty‐six percent of HPV‐positive lesions contained both alpha and beta genus subtypes (Figure 3).

**FIGURE 4:**
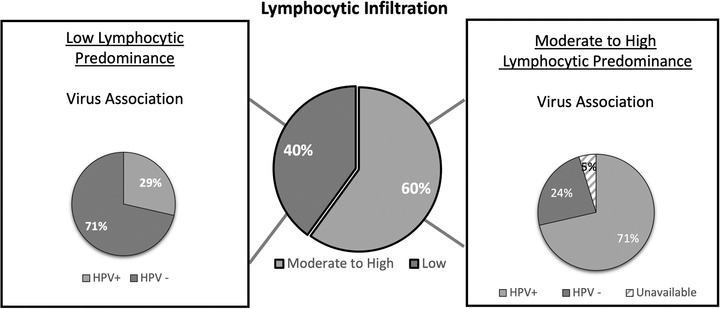
Characterization of virus genotype and lymphocytic infiltration. The majority of the lesions were characterized by dense lymphocytic infiltration. The presence of lymphocytic infiltration was significantly associated with the presence of HPV virus (*p* < 0.05)

**FIGURE 5:**
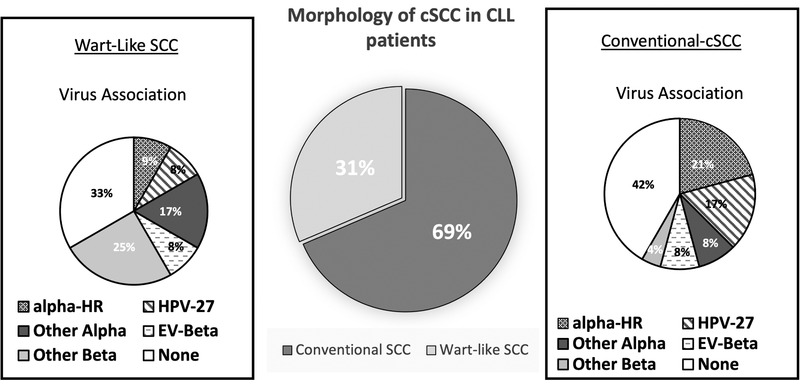
Characterization of virus genotype and histomorphology. The majority of the lesions examined were conventional cSCC, with histomorphologic features indistinguishable from those described in immunocompetent samples. More than half of the conv‐cSCC lesions contained HPV virus, with the majority representing HR‐alpha‐genus. Cutaneous SCCs with wart‐like features were described less frequently, but these lesions were equally likely to contain alpha‐ or beta‐genus subtypes. The association between HPV and histomorphology was not significant (*p* = 0.45)

Of those lesions demonstrating lymphocytic infiltration, 71% were associated with HPV infection (*p* < 0.05; Figure 4), mostly of the alpha‐genus subtype (62%; *p* = 0.11; Figure 4). There was no significant association between morphologic characteristics of the lesion (conventional vs. wart‐like morphologic features) and lymphocytic infiltration (*p* = 0.47) or HPV status (*p* = 0.45). Interestingly, 21% of the conv‐cSCC were associated with HR‐alpha‐genus viral subtypes, followed by verrucae‐associated HPV‐27 in 17% of samples examined. Cutaneous SCCs with wart‐like features were more likely to display virus association, with 67% of the samples analyzed showing HPV positivity. In contrast to conv‐cSCC, the cSCC‐wf lesions were equally likely to display alpha or beta genus specificity. HR‐HPV and HPV‐27 virus associations were less likely in cSCC‐wf (Figure 5).

Twenty‐three lesions (66% of the tumor samples) belonged to patients who did not receive chemotherapy, while 12 lesions (34% of the tumor samples) belonged to patients treated with chemotherapy. A majority of the lesions in both groups were positive for HPV, 52% in the nontreatment group and 58% in the treatment group. One of the patients had both HPV‐positive and HPV‐negative lesions, with a majority of the HPV‐positive lesions being noted after treatment with chemotherapy. One of the chemotherapy‐treated patients displayed HPV‐negative lesions; this patient was treated with anti‐CD20 therapy, while the remaining patients received purine analogues, combination therapy with anti‐CD20/purine analogue, or a BTK inhibitor.

**FIGURE 6:**
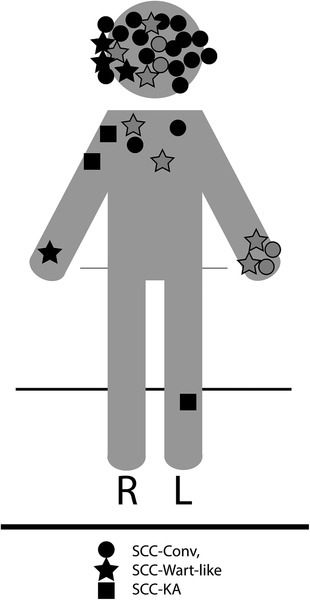
Distribution of lesions by morphologic subtype of cSCC. Lesions are predominantly in sun‐exposed locations. Circles denote conv‐cSCC, squares denote cSCC with keratoacanthomatous (KA) features, and stars denote cSCC with wart‐like features (cSCC‐wf). Shaded‐in shapes denote anterior lesions, while outlines (no shade) denote posterior lesions

There was a preponderance of HPV‐16/18 alpha genus infections found in our study cohort but interestingly, these were not found in the anogenital regions where they are typically described. In our CLL cohort, the bulk of the SCC lesions were predominantly found in sun‐exposed areas (Figure 6), including the face, arms, chest, and legs.

## 4 DISCUSSION

It is well established that patients with CLL have a higher risk of secondary malignancies than the general population, predominantly skin cancers of keratinocyte origin. Studies have reported atypical clinical presentation of cutaneous malignancy in patients with CLL [18]. We describe the histomorphology of cSCC in a cohort of CLL patients in whom the hematologic diagnosis was well established. In our cohort, the majority of the lesions displayed the morphologic variation described in cSCC exhibited by the general population, including KA features. Of specific interest, one third of the total samples examined exhibited hybrid lesions with wart‐like features (cSCC‐wf), which are not typically described in the immunocompetent population but have been well described in immunocompromised patients, such as those with BRAF‐inhibitor targeted therapy [7].

In immunosuppressed patients, HPV viruses of the beta genus contribute to the pathogenesis of cSCC [8, 19] and are associated with cSCC with wart‐like features [7]. We were interested in characterizing which virus genotypes were associated with cSCCs in CLL patients. Since cSCC‐wf have been primarily described in immunocompromised populations, we hypothesized that the majority of cutaneous HPV would be found in these lesions, with the predominant genotype being beta‐HPV.

In our cohort, more than 50% of the lesions contained HPV DNA, suggesting viral contribution to cSCC pathology in CLL. We found that a majority of both conv‐cSCC and cSCC‐wf were involved by HPV, 58% and 67%, respectively. In previous studies, beta genus HPV‐15 and 38 comprised 50% of the virus infection found in skin lesions in the CLL population [20]. We expected to find a predominance of beta‐genus HPV in our cohort as well, specifically EV‐beta genus HPV, which is considered critical in cSCC pathophysiology of transplant patients [8]. Yet, our results yielded infrequent infections by EV‐beta genus HPV in CLL patients. Only a small number of conv‐cSCC exhibited beta‐genus HPV virus. Interestingly, the predominant virus expressed in these lesions was of the alpha genus subtype, with 21% of the lesions expressing HR genotypes: HPV‐16, ‐18, and ‐56. Alpha‐ and beta‐HPV genus subtype infections were equally frequent in lesions with wart‐like features. Alpha‐genus HPV‐27, frequently associated with verrucae (warts), was more frequently described in conv‐cSCC lacking morphologic features of warts. In CLL patients, both alpha‐ and beta‐genus HPV are equally likely to contribute to the pathogenesis of cSCC.

All the cSCC lesions described in this study occurred in sun‐exposed areas. This was of particular interest since several of the lesions contained HR‐HPV, specifically HPV‐16 and ‐18, both of which are characteristically described as occurring in anogenital skin. It is well accepted that in immunocompetent patients, UV damage constitutes the main pathogenic driver for cSCC. Despite HPV infection and immunocompromised state, UV damage remains an important contributor of cSCC pathogenicity in CLL patients.

Lymphocytic infiltrates have been previously described in the cSCC of CLL patients [21]. In our cohort, 60% of the lesions had moderate to high lymphocytic infiltration, a higher frequency than what is reported in the literature [22]. Significantly, the majority of the lesions with lymphocytic infiltration also contained HPV. The primary reason for the increased incidence of skin cancer in patients with CLL is hypothesized to be the underlying impaired cellular and humoral immunity [9]. Although CLL is characterized by defective B‐lymphocytes, immune defects can be generalized beyond impaired humoral immunity. Defective CD8+ and CD4+ T‐cell function and increased regulatory T‐cell numbers have been described in CLL [10, 23], with several of these further exacerbated by existing treatment modalities, particularly the use of purine analogue, bendamustine combination, or alemtuzumab [5]. Previous studies have described that time to development of cSCC in CLL patients can be shortened by chemotherapy [5]. Despite small sample size, this trend was also noted in our study sample and reinforces the notion that T‐cell immunosuppression by CLL chemotherapy treatments contributes to cSCC. The high predominance of lymphocytes in pathology samples suggests that an inflammatory response is important to the pathophysiology of cSCC in CLL patients and possibly facilitates a role for HPV in cutaneous malignancy.

Taken together, we propose that a diagnosis of CLL leads to a dysfunctional immune system, with both elements of humoral and cellular immunity affected [9, 10, 23]. This leads to a dysregulation in tumor surveillance mechanisms that facilitates viral and UV damage‐mediated carcinogenesis. Recent studies have proposed that patients able to elicit spontaneous memory T‐cell responses against CLL‐associated antigens may have protection against the development of cutaneous skin malignancies [24]. Hence, the composition of the lymphocytic infiltrate in the cutaneous lesions may be the key to understanding the contribution of HPV viruses and UV damage to cSCC in CLL patients. It may explain the variable morphologic features of conventional versus wart‐like features, as well as the tumorigenic role of the expressed HPV subtypes, as facilitators of the accumulation of UV‐induced DNA mutations [19] versus direct agents of DNA mutagenesis.

A major strength of this study is the detailed histomorphologic description of cSCC in CLL, as well as characterization of virus genus and subtypes. Although the incidence of cSCC and prevalence of HPV in cSCC has been described in CLL patients, histomorphologic characterization has been limited. Moreover, we performed highly sensitive methodology to characterize HPV association. Although we report greater variability in genus subtypes than other studies, we also report that 43% of the lesions examined did not contain any virus, supporting a robust, high‐yield methodology with low cross contamination. Like other studies of its kind, a limitation of this study is the small sample size, comprised exclusively by Caucasian males, making generalizability of our findings difficult. An additional limitation was our inability to phenotype the lymphocytic infiltration to determine whether a characteristic signature of inflammatory cells predicts the prevalence of cSCC or genus‐specific HPV association. Despite these limitations, it is important to note that some of the trends described in prior studies with larger sample size were also observed here; specifically, reduced time to cSCC after chemotherapy treatment and lymphocytic infiltration of CLL lesions. These limitations can be mitigated in future studies by drawing from a non‐VA cohort that would include more females and a more ethnically diverse population, as well as revising the scope of pathology review to include flow cytometry.

Newer therapies have improved progression free‐ and overall survival in the CLL population. As a result, secondary malignancies, such as cSCC, are likely to become important comorbidities in long‐term outcomes of CLL patients. A better understanding of the risk factors contributing to cSCC in this population would be important as we manage long‐term complications of disease and therapy. A confirmation of the role of HPV, particularly HR‐HPV, could lead to practice changing recommendations, such as a newly defined role for vaccinations in reducing HPV‐associated malignancies.

## AUTHOR CONTRIBUTIONS

PH collected and annotated the clinical data. MH, SM, BK, and DNC performed pathology. QH, ST, and PR conducted HPV DNA assays. MH and PH performed analysis of data. GR and IRS conducted statistical analysis. GR, DNC, and IRS designed and supervised the research study and interpretation of data. IRS wrote the manuscript. All authors reviewed and approved the final text.

## CONFLICT OF INTEREST

The authors declare that there is no conflict of interest that could be perceived as prejudicing the impartiality of the research reported.

## FUNDING

IRS was supported by the American Heart Association FTF‐17400011.
